# Effectiveness and safety of Linggui Zhugan decoction for the treatment of premature contraction in patients with coronary heart disease: A systematic review and meta-analysis

**DOI:** 10.3389/fcvm.2022.1002378

**Published:** 2022-11-03

**Authors:** Longkun Liu, Yan Zhao, Yoann Birling, Yuxin Sun, Qinghua Shang, Zhong-Jing Hu, Jianping Liu, Zhaolan Liu

**Affiliations:** ^1^School of Traditional Chinese Medicine, Beijing University of Chinese Medicine, Beijing, China; ^2^Beijing University of Chinese Medicine Third Affiliated Hospital, Beijing University of Chinese Medicine, Beijing, China; ^3^NICM Health Research Institute, Western Sydney University, Penrith, NSW, Australia; ^4^Centre for Evidence-Based Chinese Medicine, Beijing University of Chinese Medicine, Beijing, China; ^5^Xiyuan Hospital of Chinese Academy of Traditional Chinese Medicine, Beijing, China; ^6^Department of Cardiovascular Medicine, Research Center of Integrated Traditional Chinese and Western Medicine, The TCM Affiliated Hospital of Southwest Medical University, Luzhou, China

**Keywords:** cardiac arrhythmia, premature contraction, Linggui Zhugan decoction, meta-analysis, effectiveness and safety, traditional Chinese medicine

## Abstract

**Objective:**

To evaluate the effectiveness and safety of Linggui Zhugan decoction (LZD) as an adjunct treatment of premature contraction in patients with coronary heart disease.

**Methods:**

PubMed, Embase, Web of Science, ClinicalTrials.gov Cochrane Library, Chinese Knowledge Infrastructure, Wanfang database, Sino Med, and VIP database were searched from inception until July 2022. Two reviewers independently selected randomized controlled trials assessing the effectiveness of LZD combined with conventional antiarrhythmic drugs in treating premature contraction in patients with coronary heart disease compared to conventional antiarrhythmic drugs only. The clinical effectiveness was considered as the primary outcome, and the times of premature junctional beats in 24 h after treatment along with adverse reactions were considered secondary outcomes. The Cochrane risk of bias 2 tool was used for the risk of bias assessment. Meta-analysis was conducted using RevMan 5.4.1. and RStudio software.

**Results:**

A total of 14 studies including 1,236 participants were included. The primary outcome indicated that, compared with antiarrhythmic drugs alone (especially β receptor blockers), the combination of LZD and conventional antiarrhythmic drugs resulted in higher clinical effectiveness (RR = 1.29, 95% CI: [1.22,1.36]) and lower number of premature junctional beats in 24 h (MD = −71.14, 95% CI: [−76.23, −66.06]) at end-of-intervention. The differences in adverse reactions (RR = 0.42, 95%CI: [0.15, 1.14], *p* = 0.09) were not significant. The risk of bias was marginally high among the studies. Funnel plot and Harbord’s test (*t* = 1.63, *p* = 0.1346) indicated no existence of publication bias.

**Conclusion:**

The current evidence shows that LZD can increase the effectiveness of conventional antiarrhythmic drugs for treating premature contraction in patients with coronary heart disease. However, the results should be interpreted with caution because of the high overall risk of bias. Future studies with appropriate randomization and double-blind methods are warranted to confirm these findings.

**Systematic review registration:**

[https://www.crd.york.ac.uk/prospero/display_record.php?RecordID=296628], identifier [CRD42022296628].

## Introduction

Premature contraction is one of the most common and severe complications of coronary heart disease (CHD). According to statistics, 60% of deaths from cardiovascular disease are due to sudden cardiac death and various kinds of cardiac arrhythmias (1). Moreover, the symptom of common heart inflammation such as myocarditis can always change from an asymptomatic presentation to a manifestation of arrhythmias (2). When the signal of premature beat fails to spread downward, cardiac output will be significantly reduced, resulting in symptoms such as dizziness, palpitations, sensation of fast heartbeat, and feeling of weakness (3, 4). Furthermore, frequent premature contraction may cause more severe arrhythmias, such as tachycardia and ventricular fibrillation (4), meanwhile, deteriorated arrhythmias in CHD will cause left ventricular systolic dysfunction, which can aggravate pre-existing CHD and eventually result in heart failure or sudden cardiac death (5, 6). Antiarrhythmic drugs, electrical therapies such as defibrillation, ablation and pacemaker implantation are all clinical treatments for premature contraction. The classification system of conventional antiarrhythmic drugs proposed by Vaughan–Williams 1975 includes Class I: sodium-channel blockers: (quinidine, lidocaine), Class II:β receptor blockers (propranolol and metoprolol), Class III: potassium-channel blockers (amiodarone and dronedarone), and Class IV: calcium-channel blockers (verapamil and diltiazem) (7–9). However, the efficacy (ability to maintain normal sinus rhythm) of current antiarrhythmic drugs is between 54 and 80% (8), which is still suboptimal. Also, large-dose use can provoke adverse reactions, especially in the gastrointestinal and neurological systems (7). In addition, the sodium-channel blockers can cause areas of slow conduction or unidirectional block in normal myocardium (10). Electrical therapies and cardioversion are significant anti-arrhythmia interventions. However, the study has shown that multiple electrical defibrillations can lead to cardiomyocyte and myocardial skeleton edema, which can be detected by T1 and T2 mapping (11). Therefore, a more effective, safer, and more moderate treatment for premature contraction is needed.

Nowadays, especially in Asian countries, a growing number of people are using Chinese herbal medicine to treat cardiovascular diseases (12). Linggui Zhugan decoction (LZD), a decoction originating from the classical text Shanghan Lun written by Zhang Zhongjing, has been used by traditional Chinese medicine practitioners to treat numerous symptoms of cardiovascular diseases for thousands of years. According to traditional Chinese medicine (13), premature contraction in coronary heart disease can be caused by heart yang deficiency and excess cold water. LZD has functions of warming yang, transforming qi, and dredging the excessive water in the body. As the upward retrograding water is regulated, yang in the heart can warm up again and the pumping function of the heart can be restored. Modern pharmacological research reveals that the constituent herbs of LZD, Cinnamomum cassia Presl (Guizhi) has cardiovascular immunoregulation and cardiovascular protective effects (14), and Rhizoma Atractylodis Macrocephalae (Baizhu) has anti-atherosclerotic, hypolipidemic, and immunomodulatory effects (15), which can be used for treating premature contraction in CHD.

Existing studies have shown that LZD combined with a low-calorie diet can reduce the risk of developing cardiovascular diseases in patients with obesity (16), and is effective in chronic heart failure (17). In recent years, research has proved that LZD has effects of promoting diuresis, antagonizing Ang II, ET-1, BNP (18), as well as reducing cholesterol and blood lipid to inhibit the occurrence or deterioration of arrhythmia in coronary heart disease. In Li’s study (19), LZD was found to be capable of treating chronic premature contraction by demonstrating its ability to regulate nuclear factors and apoptosis factors. In addition (20), the emergence of non-excitable cells and in-excitable obstacles has been identified as dominant factors in explaining the mechanism of premature contraction, which is mainly caused by myocardial tissue remodeling triggered by damages, such as CHD. This structural remodeling may eventually result in excitation–contraction couplings disorder, changes in the ion permeability of cardiomyocytes, and obstacles in cardiac excitation conduction (20, 21). These disorders can cause the heart unable to maintain regular and coordinated contraction, thus initiating premature contraction, which is characterized by high heart rate as well as the reduction in left ventricular output and cardiac output in clinical examination (2). Moreover, previous research has proved the significant curative effect of herbal medicine in the treatment of premature contraction (22), a disease that currently lacks effective medical therapy (8). We expect that LZD could reduce the occurrence of premature contraction by its functions of alleviating myocardial tissue remodeling (23), thus restoring normal cardiac contraction and sinus rhythm.

However, the evidence of the effectiveness and safety of LZD as an adjunct treatment for cardiac premature contraction in CHD has not been systematically reviewed yet. In order to provide guidance to clinicians for decision-making, we combined the results in a meta-analysis and conducted a systematic review in which the randomized controlled trials (RCTs) on LZD for premature contraction in CHD were systematically assessed.

## Materials and methods

This meta-analysis was conducted referring to the guidelines provided by Preferred Reporting Items for Systematic Review (PRISMA) 2020 and Cochrane Handbook for Systematic Reviews of Interventions (24, 25). The protocol of this systematic review and meta-analysis has been registered in PROSPERO and the registration number is CRD42022296628.

### Searches strategy

A total of nine databases were comprehensively retrieved before 21 July 2022, including PubMed, Embase, ClinicalTrials.gov Web of Science, Cochrane Library, Chinese Knowledge Infrastructure (CNKI), Sino Med, VIP, and Wanfang. Moreover, eligible studies published in peer-reviewed journals that were not found in the above databases were also included. There were no language and publication date restrictions. The following Medical Sub Headings Terms (MeSH Terms) or Title/Abstract were applied for retrieving: (Lingguizhugan decoction OR Linggui Zhugan decoction OR LZD) AND (premature contraction OR cardiac arrhythmia OR arrhythmia OR dysrhythmia) AND (coronary disease OR coronary heart disease) AND (antipremature drugs OR antiarrhythmic drugs OR metoprolol). The detailed search strategy in PubMed is shown in Table 1. The detailed search strategy in other 8 databases is shown in Appendix Tables 1–8 in the Appendix section.

**TABLE 1:** MeSH terms and title/abstract in search strategy applied to PubMed.

Items	Search terms
1	Lingguizhugan decoction [Title/Abstract] OR Linggui Zhugan decoction [Title/Abstract] OR LZD [Title/Abstract]
2	Lingguizhugan decoction [MeSH Terms] OR Linggui Zhugan decoction [MeSH Terms] OR LZD [MeSH Terms]
3	Premature Contraction [MeSH Terms] OR Arrhythmia [Title/Abstract] OR Cardiac Arrhythmia [Title/Abstract] OR Dysrhythmia [Title/Abstract]
4	Premature Contraction [MeSH Terms] OR Arrhythmia [MeSH Terms] OR Cardiac Arrhythmia [MeSH Terms] OR Dysrhythmia [MeSH Terms]
5	Coronary Disease [Title/Abstract] OR Coronary Heart Disease [Title/Abstract]
6	Coronary Disease [MeSH Terms] OR Coronary Heart Disease [MeSH Terms]
7	Antipremature drugs OR antiarrhythmic drugs OR metoprolol [Title/Abstract]
8	Antipremature drugs OR antiarrhythmic drugs OR metoprolol [MeSH Terms]
9	1 OR 2 AND 3 OR 4 AND 5 OR 6 AND 7 OR 8

### Eligibility criteria

#### Types of studies

We selected RCTs assessing the effectiveness or safety of LZD for the treatment of premature contraction in patients with CHD. The experimental and control intervention guaranteed to have the same effect should be evenly and randomly assigned to the participants. Only studies written in English or Chinese were included.

#### Participants

Participants diagnosed with coronary heart disease combined with premature contraction by explicit diagnostic standards were included. Diagnoses or symptoms of participants in the included studies should be in compliance with established Guidelines (26). In addition, there were no age, race, and region limitations.

#### Experimental intervention

The intervention groups were treated with LZD plus conventional antiarrhythmic drugs, with or without modifications. Studies using LZD combined with another decoction were excluded.

#### Control group

Studies in which the control group was treated with conventional antiarrhythmic drugs alone were included.

### Outcomes

#### Retrieval information and participants’ characteristics

We collected the following information: name of the first author’s name, publication year, sample size, participants’ age range, diagnostic criteria, dosage, and usage method of Linggui Zhugan decoction and conventional antiarrhythmic drugs, dosage and proportion of each ingredient of LZD, duration of disease, intervention duration, treatment in the intervention group and control group, outcomes evaluation measures, and results. If the data extracted by two reviewers were different, a third reviewer would be asked to determine which one was correct. We would contact the authors of the study if the data in the report were incomplete or unclear.

#### Primary outcomes

The clinical effectiveness standards in the included studies can be divided into three categories. In the accepted clinical effectiveness standard referring to (2021 Guidelines for clinical research of Traditional Chinese medicines proposed by the National Medica Products Administration, China), clinical effectiveness standards can be divided into three categories, i.e., “significantly effective,” “effective,” and “not effective.” “Significantly effective” is defined as the disappearance of clinical symptoms and reduction of 90% or more of the frequency of premature contraction (premature ventricular contraction, premature atrial, and premature junctional contraction) in the electrocardiogram. “Effective” is defined as the alleviation of clinical symptoms and reduction of 50% or more of the frequency of premature contraction in the electrocardiogram. “Not effective” is defined as a failure to meet the above criteria. In this review, we combined “significantly effective” and “effective” as a single category labeled “effective.”

#### Secondary outcomes

Additional outcomes included the number of premature junctional beats in 24 h measured with the electrocardiogram at end-of-intervention and the number of adverse reactions.

### Screening process

Retrieval results were imported into the software EndNotesX9 for screening. The screening was completed independently by two reviewers according to the search strategy. Discussions and a third reviewer’s proposal were required when the discrepancy between two reviewers emerged.

### Study risk of bias assessment

Two reviewers used the Cochrane bias risk assessment two tool to evaluate the quality of the included studies with reference to the following aspects: (a) random sequence generation, (b) allocation concealment, (c) blinding of participants and personnel, (d) blinding of outcome assessment, (e) incomplete outcome data, (f) selective reporting, and (g) other bias. Each aspect was assessed as: (a) low risk, (b) unclear risk, and (c) high risk. Discussions would be conducted when the assessments of the two reviewers were different.

### Effect measures

The risk ratio (RR) and 95% confidence interval (95%CI) of dichotomous variables (clinical effectiveness and adverse reactions) were reported and the difference between the intervention and the control groups was tested with the Mantel–Haenszel test using fixed effects. The mean difference (MD) and 95% CI of the continuous variable (number of premature junctional beats in 24 h) were reported and the difference between the intervention and the control group was tested with the inverse-variance weighting method using random effect. Overall effects with a *p*-value below 0.05 were considered statistically significant. Review manager 5.4.1 (RevMan) was used for the above analyses.

Sensitivity analysis, Labbe plot, and Baujat plot were performed using RStudio software. The quality of outcomes was assessed using Cochrane’s GRADEpro Guideline Development Tool (GDT) online software.

### Synthesis methods

#### Heterogeneity test and models

The heterogeneity test was performed by the chi-square test and it was represented by the value of I-squared. If I^2^ statistic > 50%, it can be indicated that there was a high heterogeneity and the random effects model (REM) was applied in the meta-analysis. Otherwise, the fixed effect model (FEM) was applied (27). Labbe plot was drawn to compare the therapeutic effect and individual variations between the two groups (*X*-axis represents the effect of the control group and *Y*-axis represents the effect of the intervention group) (28). In terms of continuous outcomes (29), the Baujat plot was drawn to demonstrate the contribution of each study to overall heterogeneity (*X*-axis represents the Cochran Q-test value representing the study’s contribution to the overall heterogeneity statistic and *Y*-axis represents the standardized squared difference indicating the influence of the trial). In addition, the source of heterogeneity was investigated by generalizing the results of subgroup analyses and the common characteristics of heterogeneous studies.

#### Subgroup analyses

To further explore and compare the effect of potential impactive factors on reported outcomes, subgroup analyses were conducted in accordance with the following factors: male/female ratio (male/female ratio ≥ 2, male/female ratio < 2) and duration of treatment (treatment duration < 1 month, treatment duration ≥ 1 month, treatment duration not clear).

#### Publication bias analysis

Funnel plot and Harbord’s test (linear regression test of funnel plot asymmetry) (30) were performed to evaluate potential publication bias.

## Results

### Study selection

**FIGURE 1:**
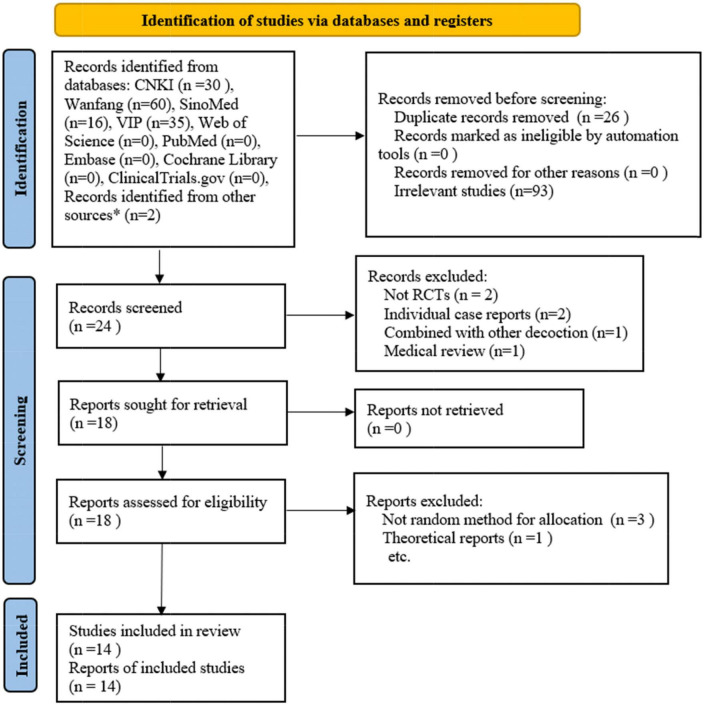
Flow plot of the study selection process. * refers to studies identified from individual journals (Medicine and health, DA JIAN KANG). RCTs: randomized controlled trials. (Template for the flow plot was provided by PRISMA).

A total of 50 references, all published in Chinese, were retrieved from the databases and two individual journals (Medicine and Health, Da Jian Kang). After removing duplicates and screening the references based on the title and abstract, the full text of the remaining 18 studies was downloaded. After the full-text screening, 14 studies were finally included in the meta-analysis. The detailed process of studies screening is shown in the PRISMA studies flowchart (Figure 1) (25).

### Study characteristics

We enrolled a total of 1,236 participants aged from 38 to 79 years including 618 participants in the control group and 618 participants in the intervention group. Clinical effectiveness was assessed in 14 studies, including 12 using the clinical effectiveness standard we accepted (i.e., “Effective”: clinical symptoms alleviated and the frequency of premature contraction in electrocardiogram was reduced by 50% or more. “Not effective”: failed to meet the above criteria) and two using other standards. The number of premature junctional beats in 24 h was assessed in seven studies (31–37), and the number of participants with adverse reactions was assessed in two studies (see Table 2) (36, 38). A total of six studies elucidated that their diagnoses were accorded with the guidelines proposed by (International Consensus on Cardiopulmonary Resuscitation and Emergency Cardiovascular Care Science With Treatment Recommendations) (26, 33, 36, 37, 39–41). In other eight studies, participants’ symptoms also met the criteria. In 12 studies, LZD was not modified in 12 studies, while two studies used modified LZD. The detailed characteristics of the included studies are shown in Table 2.

**TABLE 2:** Summary of the characteristics in each included studies.

References	Age [years] I/C	Sample size (I/C)	Duration of disease [years] I/C	Male/ Female	Treatment duration [days]	Experimental intervention (constituent herbs [g] and Administration)	Control intervention (dosage [mg] and administration)	Outcome
Chen (34)	I 54–72	78(39/39)	I 1–5	43/35	28	LZD (33, bid) + control	Metoprolol (6.25–25, bid)	➀ ➁
	C 55–72		C 1–4					
An (43)	I 42–71	80(40/40)	I 1–12	46/34	NM	LZD (30, bid) + control	Metoprolol (6.25–25, bid)	➀ ➁
	C 41–73		C 1–14					
Zhang (31)	I 68.36 ± 6.98	80(40/40)	I 4.98 ± 0.98	58/22	28	LZD (41, bid) + control	Metoprolol (6.25–25, bid)	➀ ➁
	C 69.02 ± 7.15		C 4.86 ± 0.73					
Chen (39)	39–64	100(50/50)	1–17	54/46	31	LZD (38, bid) + control	Metoprolol (25, bid)	➀
Huang (35)	40–79	86(43/43)	0.5–14	44/42	28	LZD (33, bid) + control	Metoprolol (6.25–25, bid)	➀ ➁
Xiang (37)	38–66	62(31/31)	NM	48/14	31	LZD (38, bid) + control	Metoprolol (25, bid)	➀ ➁
Li (32)	I 43–76	86(43/43)	I 1–13	51/35	28	LZD (33, bid) + control	Metoprolol (NM)	➀ ➁
	C 45–74		C 2–11					
Zheng et al. (33)	39–78	128(64/64)	0.25–16	65/63	28	LZD (33, bid) + control	Metoprolol (6.25–31.25, bid)	➀ ➁
Zhang (42)	I 61.35 ± 5.74	78(39/39)	NM	41/37	31	LZD (38, bid) + control	Metoprolol (25, bid)	➀ ➁
	C 61.66 ± 5.33							
Ding (44)	I 47–81	90(45/45)	NM	50/40	NM	LZD (41, bid) + control	Metoprolol (NM)	➀
	C 46–79							
Guo (41)	41–76	88(44/44)	NM	51/37	31	LZD (38, bid) + control	Metoprolol (25, bid)	➀ ➁
Xu (38)	I 40–66	100(50/50)	I 1–18	57/43	35	LZD (35, bid) + control	Metoprolol (6.25–25, bid)	➀ ➂
	C 39–65		C 1–16					
Zhang (36)	I 55.2 ± 6.8	90(45/45)	I 1–12	51/39	NM	Modified LZD (bid) + control	Metoprolol (25, bid)	➀ ➁ ➂
	C 55.4 ± 6.5		C 1–10					
Liu (40)	I 52.27 ± 5.33	90(45/45)	I 1–18	55/35	28	Modified LZD (bid) + control	Metoprolol (6.25–25, bid)	➀
	C 53.63 ± 5.18		C 1–16					

I, intervention group; C, control group; NM, not mentioned; bid, two times a day, LZD, Linggui Zhugan decoction, ➀: clinical effectiveness, ➁: premature junctional beats, ➂: adverse reaction.

### Risk of bias in included studies

**FIGURE 2:**
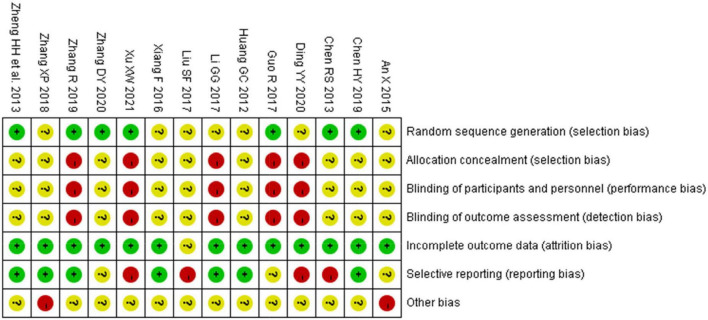
Risk of bias summary of 14 included RCTs evaluated by two reviewers independently. “ + ”: low risk, “?”: unclear risk, and “-”: high risk.

Seven studies that mentioned random number method as a randomization method (33, 34, 36, 38, 39, 41, 42) were considered to have “low risk of bias.” Seven other studies were considered to have “unclear risk of bias” in terms of “random sequence generation” (Figure 2) (32, 35–37, 40, 43, 44). In five studies (32, 36, 38, 41, 44), “Blinding of outcome assessment” and “Blinding of participants and personnel” were assessed as “high risk” due to the faulty concealment method for participants and operators, and the risk of “allocation concealment” was assessed as “high risk” due to the informed randomization method. In the aspect of “incomplete outcome data,” one study was considered to have “unclear risk” (40). Furthermore, four studies (32, 38, 39, 44) were considered to have “high risk” in “selective reporting” because they reported clinical effectiveness only, and three studies (41–43) were evaluated as “unclear risk” as the authors did not report a reduction in premature junctional beats. In two studies (31, 43), the proportion of male and female participants was significantly different between the two groups; therefore, these studies were considered to have a “high risk” in other biases.

### Clinical outcomes

#### Clinical effectiveness

**FIGURE 3:**
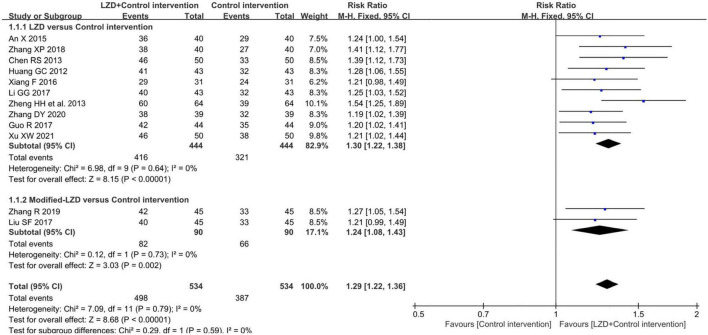
Forest plot for the comparison of clinical effectiveness between the intervention group and control group.

**FIGURE 4:**
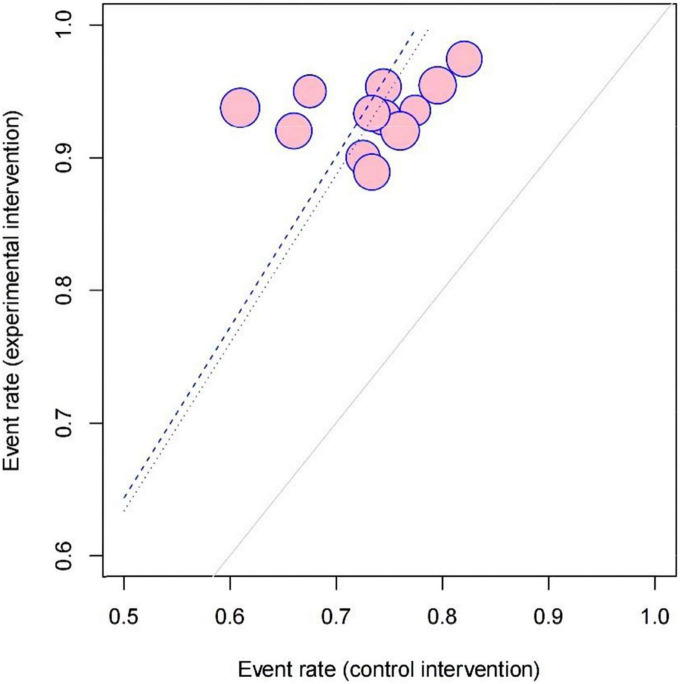
Labbe plot of evaluating the source of heterogeneity and comparing the risks stemming from the clinical effectiveness between the intervention group and control group in 12 included studies.

Twelve studies measuring clinical effectiveness according to the standard (clinical symptoms were relieved and the frequency of premature contraction in electrocardiogram was reduced by more than 50%) were combined in a meta-analysis (Figure 3). The meta-analysis showed a better clinical effectiveness in the intervention group (RR = 1.29, 95% CI: [1.22, 1.36], *P* < 0.01) compared to the control group. The heterogeneity test showed the absence of heterogeneity (χ2 = 7.09, *p* = 0.79, *I*^2^ = 0%), and the inverted triangle distribution of scatter points in the Labbe plot indicated that the experimental intervention had fewer individual variations in increasing clinical effectiveness compared to the control intervention (Figure 4).

#### Reduction in premature junctional beats

**FIGURE 5:**
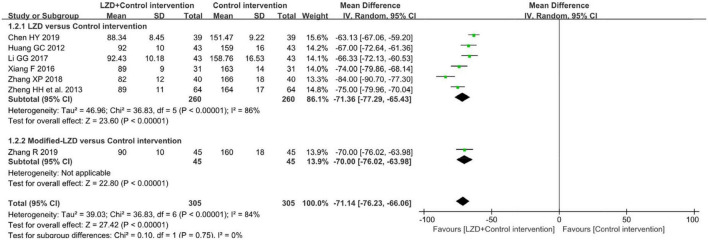
Forest plot of the between-group for the comparison of the times of premature junctional beats in 24 h after treatment.

The results of the meta-analysis of seven studies showed that (31–37), compared to the control group, the number of premature junctional beats was significantly lower in the intervention group (MD = −71.14, 95% CI: [−76.23, −66.06], *p* < 0.01). However, this conclusion should be treated with prudence because the Chi-square test showed significant heterogeneity among the studies (χ2 = 36.83, *p* < 0.00001, *I*^2^ = 84%) (Figure 5).

#### Subgroup analyses results

**FIGURE 6:**
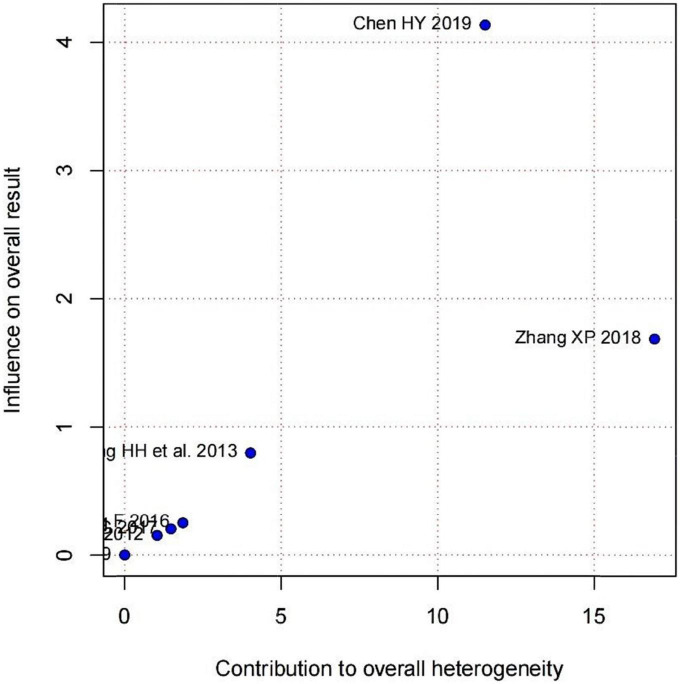
Baujat plot demonstrating each study’s contribution to overall heterogeneity from seven studies that reported premature junctional beats after treatment (X-axis represents Cochran Q-test value representing the study’s contribution to the overall heterogeneity).

Subgroup analysis in accordance with male/female ratios revealed that when male/female ratio ≥ 2, the combination of LZD and conventional antiarrhythmic drugs increased clinical effectiveness more effectively (RR = 1.31, 95%CI: [1.12, 1.54]) and times of premature junctional beats in 24h decreased more (MD = −78.86, 95%CI: [−88.66, −69.07]). In treatment duration < 1-month subgroup, the increase in clinical effectiveness was more notable (RR = 1.34, 95%CI: [1.23, 1.47]). Moreover, there was a greater decrease in times of premature junctional beats in 24h when treatment duration ≥ 1 month (MD = −74.00, 95%CI = [−79.86, −68.14]) (Table 3). According to subgroup analyses and Baujat plot (Figure 6), the high heterogeneity mainly stemmed from the studies in treatment duration < 1 month subgroup (*I*^2^ = 88%).

**TABLE 3:** Subgroup analysis of two reported outcomes according to different treatment durations and male/female ratios.

Outcomes	Sample size (I/C)	RR	MD	95%CI	*P*-value	I^2^ statics
**Clinical effectiveness**						
Male/Female (ratio) ≥ 2	142(71/71)	1.31	–	(1.12, 1.54)	0.0006	0%
Male/Female (ratio) < 2	926(463/463)	1.28	–	(1.21, 1.36)	<0.00001	0%
**Premature junctional beats after treatment**						
Male/Female (ratio) ≥ 2	142(71/71)	–	−78.86	(−88.66, −69.07)	<0.00001	79%
Male/Female (ratio) < 2	468(234/234)	–	−68.19	(−72.59, −63.80)	<0.00001	72%
**Clinical effectiveness**						
Treatment duration ≥ 1 month	428(214/214)	1.24	–	(1.14, 1.35)	<0.00001	0%
Treatment duration < 1 month	470(235/234)	1.34	–	(1.23, 1.47)	<0.00001	0%
Treatment duration not clear	170(85/85)	1.26	–	(1.09, 1.45)	0.002	0%
**Premature junctional beats after treatment**						
Treatment duration ≥ 1 month	458(229/229)	–	−74.00	(−79.86, −68.14)	<0.00001	–
Treatment duration<1 month	62(31/31)	–	−70.88	(−77.82, −63.94)	<0.00001	88%
Treatment duration not clear	90(45/45)	–	−70.00	(−76.02, −63.98)	<0.00001	–

I, intervention group; C, control group; RR, risk ratio; MD, mean difference, and CI, confidence interval.

#### Comparison of Linggui Zhugan decoction and modified Linggui Zhugan decoction with conventional antiarrhythmic drugs

As a feature of traditional Chinese medicine treatment, LZDs were accessed separately, with or without modifications. According to the results of clinical effectiveness and reduction in premature junctional beats, we found that unmodified LZD exerted better effects in increasing clinical effectiveness (RR = 1.30, 95%CI: [1.22,1.38]) and reducing premature junctional beats (MD = −71.36, 95% CI: [−77.29, −65.43]).

#### Adverse reaction

**FIGURE 7:**
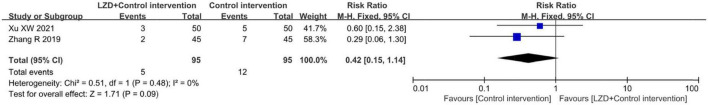
Forest plot for comparison between the intervention group and control group concerning adverse events (hypotension, nausea, bradycardia, vertigo, and fatigue) after treatment in two included studies.

Two studies (36, 38) reported adverse reactions, which included episodes of hypotension, nausea, bradycardia, vertigo, and fatigue. In these two studies, the incidence of adverse reactions was less than 6%, with the control group experiencing more adverse reactions. However, the difference was not statistically significant (RR = 0.42, 95% CI: [0.15, 1.14], *p* = 0.09) (Figure 7).

### Publication bias analysis

**FIGURE 8:**
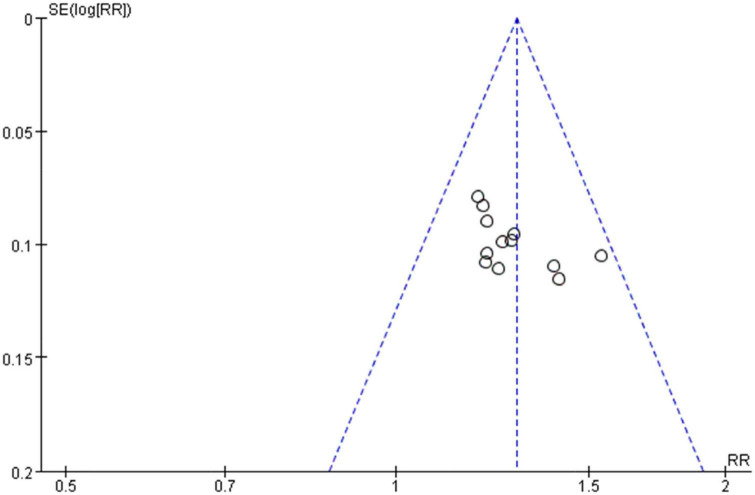
Funnel plot of the clinical effectiveness reported by 12 included studies using the accepted standard (i.e., “Effective”: clinical symptoms alleviated and the frequency of premature contraction in electrocardiogram was reduced by 50% or more. “Not effective”: failed to meet the above criteria).

A funnel plot of clinical effectiveness (fixed effect model) reported by 12 studies was drawn. The scattered points distribution in the funnel plot were basically symmetrical, and the distribution quantity on the left and right sides was basically equal. Harbord’s test was performed for further verification. The linear regression test of funnel plot asymmetry showed that there was almost no publication bias in the included studies: (*t* = 1.63, *p* = 0.1346 > 0.05) and the results were relatively reliable (Figure 8).

### Assessment of evidence quality

The quality of the evidence was presented in a summary of findings (SoF) table (Table 4). Certainty of the evidence was low in the outcomes of two subgroup analyses due to inconsistency and indirectness and it was moderate in the outcomes of clinical effectiveness and adverse reactions because of the risk of bias and large effects. Symbols in the SoF table represent: ⊕⊕⊕⊕: high, ⊕⊕⊕○: moderate, ⊕⊕○○: low, and ⊕○○○: very low.

**TABLE 4:** Summary of findings: LZD for the treatment of premature contraction in patients with coronary heart disease.

Outcome No of participants (studies)	Relative effect (95% CI)	Anticipated absolute effects (95% CI)			Certainty	What happens
		
		Control intervention	Experimental intervention	Difference		
Clinical effectiveness No of participants: 1068 (12 RCTs)	RR 1.29 (1.22 to 1.36)	72.5%	93.5% (88.4 to 98.6)	21.0% more (15.9 more to 26.1 more)	⊕⊕⊕○ Moderate ^a^	Adding LZD to conventional antiarrhythmic drug feasibly increases the clinical effectiveness
Premature junctional beats in 24 h after treatment No of participants: 610 (7 RCTs)	–	–	–	MD 71.14 lower (76.23 lower to 66.06 lower)	⊕⊕○○ Low ^b,c,^	The combination of LZD and conventional antiarrhythmic drugs creates a more effective approach in reducing premature junctional beats
Adverse reactions No of participants: 190 (2 RCTs)	RR 0.42 (0.15 to 1.12)	12.6%	5.3% (1.9 to 14.1)	7.3% fewer (10.7 fewer to 1.5 more)	⊕⊕⊕○ Moderate ^d,e^	Adding LZD to conventional antiarrhythmic drug will not increase the incidence of adverse reactions

LZD for the treatment of cardiac premature contraction in patients with coronary heart disease.

Patient or population: patients clinically diagnosed with coronary heart disease complicated with premature contraction.

Intervention: LZD + conventional antiarrhythmic drugs.

Comparison: conventional antiarrhythmic drugs.

*The risk in the intervention group (and its 95% confidence interval) is based on the assumed risk in the comparison group and the relative effect of the intervention (and its 95% CI).

CI, confidence interval; MD, mean difference; RR, risk ratio.

GRADE working group grades of evidence.

High certainty: ⊕⊕⊕⊕ we are very confident that the true effect lies close to that of the estimate of the effect.

Moderate certainty: ⊕⊕⊕○ we are moderately confident in the effect estimate: the true effect is likely to be close to the estimate of the effect, but there is a possibility that it is substantially different.

Low certainty: ⊕⊕○○ our confidence in the effect estimate is limited: the true effect may be substantially different from the estimate of the effect.

Very low certainty: ⊕○○○ we have very little confidence in the effect estimate: the true effect is likely to be substantially different from the estimate of effect.

^*a*^In 3 of the studies recording clinical effectiveness, a faulty concealment method was applied which caused a high risk of bias in “Blindings.”

^*b*^Meta-analysis for comparison of the times of premature junctional beats in 24 h revealed high heterogeneity [χ^2^ = 36.83, P < 0.00001, I^2^ = 84%], which resulted in inconsistency.

^*c*^The subgroup analysis only reported premature junctional beats as the efficacy evaluation of premature contraction which may lead to indirectness.

^*d*^In the forest plot for comparison of adverse reactions, 95% confidence interval ranges of both two included studies crossed the invalid line which may cause imprecision.

^*e*^Small sample size (*n* = 190).

## Discussion

A total of 14 studies involving 1,236 participants were included in this systematic review. We found that the combination of LZD and conventional antiarrhythmic drugs presented better clinical effectiveness and was more effective in reducing premature junctional beats compared to antiarrhythmic drugs alone. In practical clinical application, LZD can be used as an alternative therapy for patients who have poor effectiveness after using conventional antiarrhythmic drugs or cannot bear the side effects of western medicine and defibrillation.

When male/female ratio was 2 and above, the curative effect was more prominent, possibly because the experimental intervention was more efficacious to man masses, but the specific mechanism is still unclear and remains to be studied by further research. Also, we found that the times of premature junctional beats decreased obviously in the treatment duration ≥ 1-month subgroup which indicated that at least 1 month of treatment may be needed for optimal efficacy. However, the heterogeneity was high in the outcomes of premature junctional beats [*I*^2^ = 84%] which mainly came from studies with a treatment duration of less than 1 month [*I*^2^ = 88%]. Also, the scatter point distribution in the Labbe plot (Figure 3) suggested that the usage of LZD combined with conventional antiarrhythmic drugs may have less undetermined effects and individual variations in treating premature contraction. In the aspect of modifications, on the contrary, basic LZD exerted better curative effects. This indicated that the application of modifications by clinicians should be appropriate.

However, the studies we included in this study have several limitations. Given that only two studies reported adverse reactions as part of the safety evaluation and that the sample size was small, the conclusions should be interpreted with caution and should be investigated further. The studies included in this meta-analysis were of low quality, with small sample sizes, no rigorous experimental protocol, short follow-up duration, and simple effect evaluation. Because of the existing publication bias and low quality of included studies, larger sample sizes and more meticulous methodology are required more meticulous methodology are required.

This is the first systematic review that evaluates the efficacy and safety of LZD for premature contraction in patients with coronary heart disease, and it provides a more effective and safer TCM therapy for the clinical treatment of arrhythmias, particularly premature beats. The innovation of this study compared to existing meta-analyses is that we included the Labbe plot and Baujat plot to explicitly analyze the degree of contribution of each study to overall heterogeneity and evaluate the quality of each included study. In addition, we used the SoF table (Table 4) to evaluate the quality of evidence for each outcome, which can play a reference role for readers and clinical practitioners in related fields. The strength of this systematic review is incarnated in the rigorous methodology and diversified subgroup analyses covering multiple factors. In contrast to other studies, our subgroup analysis is not only to deal with heterogeneity but also to extract effective clinical conclusions for reference and study. Although it provides important guidance for clinicians and researchers, this systematic review has several deficiencies. The specific mechanism of LZD needs to be further clarified, due to the limited pharmacological research on LZD. We could only find 14 relevant studies that met the research purpose, which was one of the reasons for the significant heterogeneity. This meta-analysis provides implications for clinicians that adding LZD and conducting long-term adjunct treatment can improve clinical effectiveness with lower individual variations.

## Conclusion

Adding LZD to conventional antiarrhythmic drugs can increase the clinical effectiveness which provides more effective treatment in alleviating clinical symptoms like chest tightness, palpitation, and fatigue. Meanwhile, meta-analysis revealed that LZD combined with conventional antiarrhythmic drugs is a superior antiarrhythmic agent in reducing the occurrences of premature junctional beats. According to the results of subgroup analyses, LZD was more efficacious to man masses and the effect of reducing premature junctional beats was more prevalent by applying long-term LZD treatment.

## Data availability statement

The original contributions presented in this study are included in the article/Supplementary material, further inquiries can be directed to the corresponding author.

## Author contributions

LL conceived and designed research ideas and collected and analyzed the data. YZ and YS conducted the data collection and analysis. QS made decisions on the differences of the extracted data. YB and Z-JH proposed modification suggestions for the draft. ZL constructed the experimental design and ideas and drafted the initial manuscript with LL. All authors reviewed the draft at the end.

## Appendix

**TABLE A1:** CNKI search strategy.

Items	Search terms
1	TI = (‘心律失常’ + ‘早搏’ + ‘房性早搏’ + ‘室性早搏’ + ‘交界性早搏’ + ‘房室交界性早搏’ + ‘冠心病心律失常’ + ‘冠状动脉粥样硬化性心脏病心律失常’)
2	TI = (‘苓桂术甘汤’ + ‘苓桂术甘’)
3	TI = (‘治疗’ + ‘疗效’ + ‘临床观察’ + ‘临床评估’ + ‘临床试验’ + ‘临床效果’ + ‘临床研究’ + ‘随机’ + ‘对照’)
4	1 AND 2 AND 3
5	KY = (‘心律失常’ + ‘早搏’ + ‘房性早搏’ + ‘室性早搏’ + ‘交界性早搏’ + ‘房室交界性早搏’ + ‘冠心病心律失常’ + ‘冠状动脉粥样硬化性心脏病心律失常’)
6	KY = (‘苓桂术甘汤’ + ‘苓桂术甘’)
7	KY = (‘治疗’ + ‘疗效’ + ‘临床观察’ + ‘临床评估’ + ‘临床试验’ + ‘临床效果’ + ‘临床研究’ + ‘随机’ + ‘对照’)
8	5 AND 6 AND 7
9	4 OR 8

**TABLE A2:** Wanfang search strategy.

Items	Search terms
1	题名或关键词 = (“心律失常” or “早搏” or “房性早搏” or “室性早搏” or “交界性早搏” or “房室交界性早搏” or “冠心病心律失常” or “冠状动脉粥样硬化性心脏病心律失常”)
2	题名或关键词 = (“苓桂术甘汤” or “苓桂术甘”)
3	题名或关键词 = (“治疗” or “疗效” or “临床观察” or “临床评估” or “临床试验” or “临床效果” or “临床研究”or “随机” or “对照”)
4	1 AND 2 AND 3

**TABLE A3:** Sinomed search strategy.

Items	Search terms
1	“心律失常”[标题] OR “早搏”[标题] OR “房性早搏”[标题] OR “室性早搏”[标题] OR “交界性早搏”[标题] OR “房室交界性早搏”[标题] OR “冠心病心律失常”[标题] OR “冠状动脉粥样硬化心脏病心律失常”[标题]
2	“苓桂术甘汤”[标题] OR “苓桂术甘”[标题]
3	“治疗”[标题] OR “疗效”[标题] OR “临床观察”[标题] OR “临床评估”[标题] OR “临床试验”[标题] OR “临床效果”[标题] OR “临床研究”[标题] OR “随机”[标题] OR “对照”[标题]
4	1 AND 2 AND 3

**TABLE A4:** VIP search strategy.

Items	Search terms
1	M = 心律失常 OR 早搏 OR 房性早搏 OR 室性早搏 OR 交界性早搏 OR 房室交界性早搏 OR 冠心病心律失常 OR 冠状动脉粥样硬化性心脏病心律失常
2	M = 苓桂术甘 OR 苓桂术甘汤
3	M = 治疗 OR 疗效 OR 临床观察 OR 临床评估 OR 临床试验 OR 临床效果 OR 临床研究 OR 随机 OR 对照
4	1 AND 2 AND 3

**TABLE A5:** Cochrane library search strategy.

Items	Search terms
1	Lingguizhugan decoction [Title Abstract Keyword] OR Linggui Zhugan decoction [Title Abstract Keyword] OR LZD [Title Abstract Keyword]
2	Premature Contraction [Title Abstract Keyword] OR Arrhythmia [Title Abstract Keyword] OR Cardiac Arrhythmia [Title Abstract Keyword] OR Dysrhythmia [Title Abstract Keyword]
3	Coronary Disease [Title Abstract Keyword] OR Coronary Heart Disease [Title Abstract Keyword]
4	antipremature drugs OR antiarrhythmic drugs OR metoprolol [Title Abstract Keyword]
5	RCT OR randomized controlled trail OR clinical trial OR randomly
6	1 AND 2 AND 3 AND 4 AND 5

**TABLE A6:** Web of Science search strategy.

Items	Search terms
1	Lingguizhugan decoction [Topic] OR Linggui Zhugan decoction [Topic] OR LZD [Topic]
2	Premature Contraction [Topic] OR Arrhythmia [Topic] OR Cardiac Arrhythmia [Topic] OR Dysrhythmia [Topic]
3	Coronary Disease [Topic] OR Coronary Heart Disease [Topic]
4	antipremature drugs [Topic] OR antiarrhythmic drugs [Topic] OR metoprolol [Topic]
5	RCT OR randomized controlled trail OR clinical trial OR randomly
6	1 AND 2 AND 3 AND 4 AND 5

**TABLE A7:** Embase (Elsevier) search strategy.

Items	Search terms
1	Lingguizhugan decoction [keyword/title/subject area] OR Linggui Zhugan decoction [keyword/title/subject area] OR LZD [keyword/title/subject area]
2	Premature Contraction [keyword/title/subject area] OR Arrhythmia [keyword/title/subject area] OR Cardiac Arrhythmia [keyword/title/subject area] OR Dysrhythmia [keyword/title/subject area]
3	Coronary Disease [keyword/title/subject area] OR Coronary Heart Disease [keyword/title/subject area]
4	antipremature drugs [keyword/title/subject area] OR antiarrhythmic drugs [keyword/title/subject area] OR metoprolol [keyword/title/subject area]
5	RCT OR randomized controlled trail OR clinical trial OR randomly
6	1 AND 2 AND 3 AND 4 AND 5

**TABLE A8:** ClinicalTrials.gov search strategy.

Items	Search terms
1	Status: All studies
2	Lingguizhugan decoction OR Linggui Zhugan decoction OR LZD
3	Condition or disease: Premature Contraction OR Arrhythmia OR Cardiac Arrhythmia OR Dysrhythmia
4	Condition or disease: Coronary Disease OR Coronary Heart Disease
5	Other terms: RCT OR randomized controlled trial OR clinical trial OR randomly OR Lingguizhugan decoction OR Linggui Zhugan decoction OR LZD
6	1 AND 2 AND 3 AND 4 AND 5
